# Advancing Gastrointestinal Health: Curcumin’s Efficacy and Nanopreparations

**DOI:** 10.3390/molecules29071659

**Published:** 2024-04-07

**Authors:** Jialin Ji, Zhaojie Ma, Yingshuai Wang

**Affiliations:** 1 School of Clinical Medicine, Shandong Second Medical University, Weifang 261053, China; j700909700701@163.com; 2 School of Humanities and Medicine, Shandong Second Medical University, Weifang 261053, China; ma961005@163.com; 3 School of Life Science and Technology, Shandong Second Medical University, Weifang 261053, China

**Keywords:** curcumin, gastrointestinal disease, inflammatory bowel disease, *Helicobacter pylori* colon cancer, nanopreparations

## Abstract

Curcumin (CCM) is a polyphenol compound extracted from the turmeric rhizome. It has various biological activities, including antibacterial, anti-inflammatory, anti-cancer, and antioxidant. Due to its diverse activities, it is often used by researchers to study the therapeutic effects on various diseases. However, its poor solubility leads to poor bioavailability, and it is necessary to increase the water solubility with the help of carriers to improve the therapeutic effect. Gastrointestinal disease is a major global health problem that continues to affect human health. In this review, we have summarized the possible mechanism and therapeutic effect of CCM in various gastrointestinal diseases, and the improvement in the curative effect of CCM with nanopreparation. Finally, we concluded that there have been many clinical trials of CCM in combination with other drugs for the treatment of gastrointestinal disease, but so far, few have used CCM nanomaterials for treatment. Although in vitro and preclinical experiments have shown that nanopreparations can improve the efficacy of CCM, there are still insufficient studies on the safety of carriers.

## 1. Introduction

Gastrointestinal disease mainly refers to inflammatory gastrointestinal diseases (gastritis and inflammatory bowel disease, etc.), gastric cancer, and colon cancer. Because of its long duration, difficulty of treatment, and recurrent nature, gastrointestinal disease remains an important global health problem. The incidence rate of gastrointestinal disease is high. Recently, chronic atrophic gastritis was estimated to affect approximately 25% of the general population worldwide [1]. *Helicobacter pylori* (*H. pylori*) is one of the pathogenic factors of chronic gastritis. In Western countries, the rate of *H. pylori* infection in children is about 10%, and in developing countries, it is as high as 50% [2].

Natural products have continued to play an important role in gastrointestinal disease, but their complex chemical structure results in poor stability and solubility, posing challenges in drug development. Discovering a new dosage form is a necessary way to enhance the bioavailability of natural products in the human body. Nanomaterials have been the focus of research, which can improve the bioavailability of drugs, can be designed to target lesions, and can improve efficacy by controlling the rate of drug release [3]. Therefore, it can reduce the dosages to reduce the side effects of drugs.

Curcumin (CCM) is a polyphenolic compound extracted from the turmeric rhizome. Turmeric rhizome has been considered a food throughout history and is also a useful herb in China. It has been recorded in the *Chinese Pharmacopoeia* for treating gastrointestinal diseases. CCM has rich biological activity, such as anti-inflammation [4,5], antibacterial [6], antitumor [7], and antioxidant properties [8]. Therefore, it can be used to treat and prevent inflammatory diseases [9] and cancers [10]. However, there is no perfect cure in the world. Although CCM has promising therapeutic value, its poor solubility limits its potential to be a simple monomeric drug.

Researchers have used a variety of methods to address this challenge, such as chemical modification [11], nanotechnology [12,13], and the use of natural synergists [14]. In this review, we summarize the molecular mechanism of CCM on inflammatory bowel disease (IBD), gastric ulcers, and colon cancer, and evaluate and predict the clinical efficacy of CCM formulations based on the existing studies.

## 2. Structural Characteristics of Curcumin

**Figure 1:**
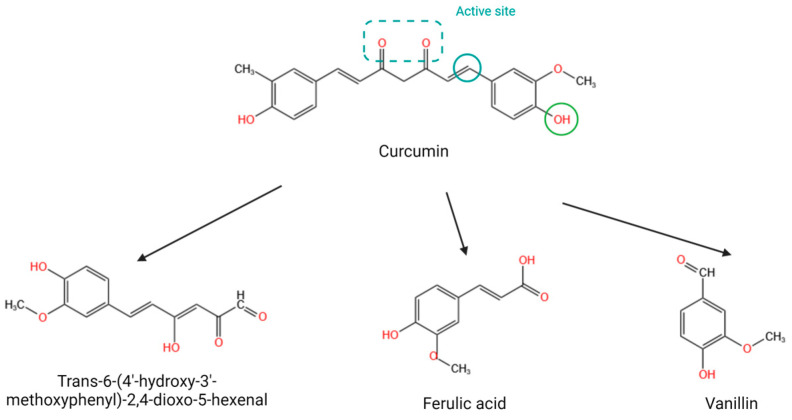
Structure, active sites, and main metabolites of CCM.

CCM is the primary active compound found in turmeric. Its complex structure provides it with a range of biological activities, but also results in low solubility, poor absorption in the gastrointestinal tract, extremely low bioavailability, and rapid metabolism and excretion [15]. CCM contains a polycyclic structure and long hydrocarbon chains (Figure 1). The presence of hydroxyl and methoxy groups in its structure cannot improve the polarity of CCM, which leads to poor solubility. The solubility of CCM is estimated to be 11 ng·mL^−1^. It is poorly absorbed by the gastrointestinal tract. Oral administration of a single dose of 2 g of curcumin resulted in a plasma concentration of less than 5 μg·mL^−1^ in rats (https://pubchem.ncbi.nlm.nih.gov/compound/curcumin, accessed on 21 March 2024). In addition, CCM is mostly excreted in the stool. Shehata et al. [16] discovered that approximately 75% of the CCM administered to rats was excreted in feces. Furthermore, CCM is easily degraded under alkaline conditions Figure 1 [17], resulting in the loss of active groups [18]. Although metabolites such as ferulic acid and vanillin possess certain biological activities, their potency is inferior to that of CCM. Wright et al. [19] showed that ferulic acid and vanillin could not inhibit the growth of breast cancer cells and the secretion of parathyroid hormone-related protein.

## 3. Bioactivity of Curcumin

### 3.1. Antibacterial

CCM has been found to inhibit the growth of a wide variety of bacteria [20], including Gram-negative and Gram-positive bacteria. According to Sharahi et al. [21], CCM is effective against extensively drug-resistant bacteria isolated from burn wound infections. The minimum inhibitory concentration levels ranged from 128 to 512 μg·mL^−1^. The MIC of CCM for *K. pneumoniae* was 128–256 μg·mL^−1^, for *P. aeruginosa* it was 128–512 μg·mL^−1^, and for *A. baumannii* it was 128–512 μg·mL^−1^. The minimum inhibitory concentration and minimum bactericidal concentration of CCM against *H. pylori* were 30 and 40 μg·mL^−1^ [22], respectively. In a mouse model of *H. pylori* infection, CCM not only eradicated *H. pylori* but also restored gastric damage. These studies prove that CCM has inhibitory effects on bacterial proliferation both in vitro and in vivo [23].

### 3.2. Regulating Gut Microbes

CCM not only inhibits bacterial growth but also regulates the gut microbiota. Studies have shown that CCM can affect the bacterial abundance of mice. Specifically, it increases the relative abundance of *Lactobacilli* and decreases the relative abundance of *Coriobacterales* [24]. In addition, CCM was able to regulate the proportion of microorganisms linked to colorectal cancer in mouse models [25]. In colitis-associated colorectal cancer mice fed with CCM-supplemented diets, McFadden et al. found that CCM significantly reduced the relative abundance of *Clostridiales* while increasing the abundance of *Lactobacillales*, *Bifidobacteriales*.

### 3.3. Anti-Inflammation and Antitumor

CCM is known for its anti-inflammatory properties. with signal transduction and transcription factors. Its ability to interact with these immune mediators also gives it anticancer activity.

CCM inhibits TNF-α transcription and the activation of the NF-κB pathway [26]. It also inhibits the phosphorylation of IκB kinase and the nuclear translocation of NF-κB [27], thereby further inhibiting the activity of the NF-κB pathway. This results in the inhibition of several pro-inflammatory cytokines, including cancer-related cytokines (IL-1, IFN-γ). Furthermore, it increases the phosphorylation of c-Jun N-terminal kinase (JNK) [28], resulting in anti-cancer effects. Studies have reported that CCM affects the anticancer activity of NF-κB and AP-1 transcription factors in vitro models [26].

In addition to regulating transcription factors, CCM also modulates inflammatory signals by affecting immune cells. For instance, it regulates the polarization balance of M1/M2 macrophage [29], Th17/Treg homeostasis [30], and memory B cells [31]. Additionally, CCM can impact the growth of cancer cells by influencing the cell cycle [32]. These studies provide strong evidence that CCM has potential as an anti-inflammatory and anti-cancer drug.

### 3.4. Antioxidant

CCM has antioxidant effects due to its structural characteristics. It contains structures such as phenolic hydroxyl, methoxyl, and 1,3 beta-dione that may neutralize reactive oxygen species (ROS) intermediates [17]. Studies have shown that CCM can up-regulate the expression level of Nfr2 protein to enhance the activity of antioxidant enzymes [33], as well as activate PPARα/γ to reduce ROS and oxidative stress response [34]. CCM protects the system from oxidative stress in various ways.

## 4. Curcumin Prevents and Treats Gastrointestinal Disease

### 4.1. Inflammatory Bowel Disease

Inflammatory bowel disease (IBD) is a chronic condition, causing inflammation in the digestive tract. It includes ulcerative colitis (UC) and Crohn’s disease (CD). UC is a gastrointestinal disease that is localized to the large intestine, or colon, where inflammation can affect either the entire organ or a portion of it. CD can affect any part of the GI tract, but most commonly involves both the large and small intestines [35]. IBD is a significant risk factor for the development of colon cancer. The patient presents with abdominal distension, abdominal pain, and diarrhea. The stool may contain watery mucus, mucous pus, pus, blood, or a combination of these. The cause of IBD is unknown, but it is believed to be the result of a combination of genetic, immune, environmental factors, and gut microbes [36]. In the treatment of IBD with traditional drugs, adverse reactions are common. Therefore, it is necessary to find an effective and safe drug for adjuvant therapy. CCM has been studied extensively due to its ability to regulate inflammatory signals and gut microbes, making it a potential candidate for preventing and treating inflammatory bowel disease.

Multiple pathways regulate inflammatory signaling in IBD, including NF-κB. CCM has been shown to reduce the expression of TNF-α and inhibit the activation of the NF-κB pathway. It also regulates the NF-κB/IκB pathway, reducing the levels of downstream proinflammatory cytokines such as IL-1, IL-6, and IL-23. Additionally, in colon epithelial cells stimulated with 100 U·mL^−1^ IFN-γ for 5 h, 50 μM CCM can inhibit IFN-γ signal in the cells [37], leading to reduced inflammation levels in the colon. CCM can alleviate DSS-induced colitis in mice by modulating memory B cells and the Bcl-6-Syk-BLNK pathway. Wei et al. [31] (2022) found that after oral CCM (100 mg/kg/d) for 14 consecutive days in mice with DSS-induced colitis, the levels of CD19CD27 memory B cells in the peripheral blood of colitis mice were significantly reduced, and the expressions of Bcl-6, CIN85, Syk, and p-Syk in colonic tissue were significantly inhibited.

CCM is effective in various colitis models, including those induced by dinitrobenzene sulfonic acid (DNBS), 2,4-dinitrochlorobenzene (DNCB), and DSS [9]. B. Salh et al. [38] (2003) used a DNB-induced colitis mouse model to test the anti-inflammatory effects of CCM by adding CCM to the mouse diet at a concentration of 0.25% after 5 days of DNB instillation. The results showed that CCM can alleviate experimental colitis by inhibiting IL-1β expression, p38 MAPK and NF-κB activation. Camacho-Barquero, L. et al. [39] (2007) induced colitis in rats using trinitrobenzensulfonic acid (30 mg/animal) and oral treatment with CCM (50 or 100 mg/kg/day). Consistent with the results of B. Salh et al., CCM can reduce the activation of p38 MAPK. Additionally, CCM enhances gut barrier function by regulating gut microbiome homeostasis. For instance, CCM increased the relative abundance of bacteria-producing butyrate in mice, increasing butyrate, which is beneficial for gut homeostasis [40].

Clinical trials have demonstrated the safety and efficacy of CCM in treating IBD, including ulcerative colitis and Crohn’s disease [9]. Subjects treated with CCM alone or in combination with other drugs experienced varying levels of relief compared with placebo. In a meta-analysis by Zeng et al. [41], the efficacy and safety of CCM for UC were evaluated. The analysis comprised 9 randomized controlled trials (RCTs) that included 507 patients with mild to moderate ulcerative colitis Table 1. The efficacy and safety of CCM in UC treatment needs to be further verified by more RCTs, although CCM may improve activity index, clinical response, and endoscopic response in UC patients, and reduce ESR and CRP, these findings require further confirmation.

### 4.2. Gastrointestinal Disease Caused by Helicobacter pylori

*H. pylori* infection is a common global issue. Although the rate of infection has decreased significantly in recent years [55], it still affects almost half of the world’s population [56]. This bacterium is highly associated with duodenal ulcers, gastric ulcers, and gastric cancer. The emergence of clarithromycin-resistant *H. pylori* [57] has led to the development of natural antibiotics.

**Figure 2:**
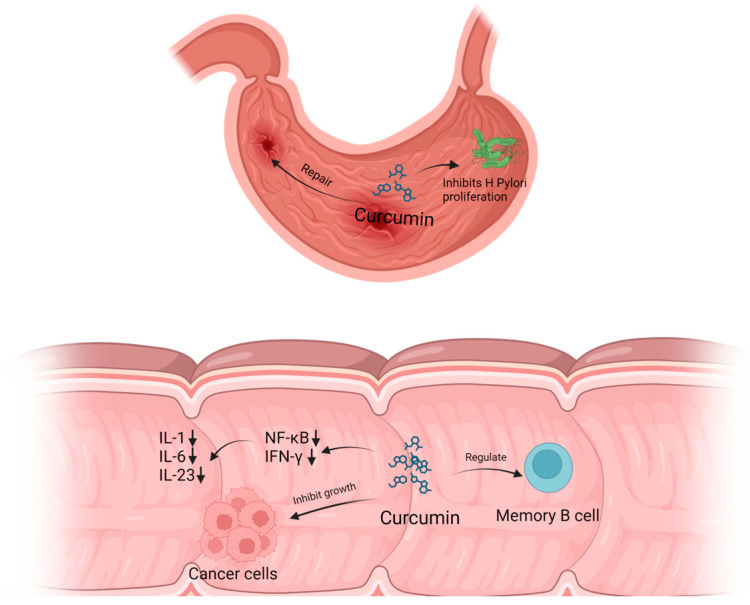
Mechanism of CCM in the treatment of gastrointestinal disease.

Ronita De et al. [39] administered CCM (25 mg/kg/d) orally to *H. pylori*. infected mice for seven consecutive days, and the results showed that CCM could eradicate *H. pylori*. in the stomach of mice, confirming the anti-*H. pylori*. potential of CCM Figure 2. Ashwini Kumar Ray et al. [58] co-cultured *H. pylori*. with 40 μM CCM phospholipid complexed formulation and found that the formation of bicyclopentadione, one of the stable end products of CCM oxidative transformation, was related to the growth inhibition of *H. pylori*. by curcumin. After co-culture for 6 h, 20 μM CCM phospholipid complex inhibited *H. pylori* CagA translocation and c-Src phosphorylation. In addition, Ashwini Kumar Ray et al. treated gastric epithelial cells infected with *H. pylori* with CCM stabilizing acetalcurcumin and diacetalcurcumin. The results showed that the oxidative transformation of curcumin was necessary to reduce *H. pylori* infection response, which provided mechanism evidence for CCM resistance to *H. pylori*.

Two recent clinical trials Table 1 have demonstrated that CCM can reduce inflammation in patients with chronic gastritis caused by *H. pylori* [51,52]. In both studies, CCM was added to traditional triple therapy and compared to a group receiving only triple therapy. Judaki, A. et al. used omeprazole, amoxicillin, and metronidazole, while Khonche, A. et al. used clarithromycin, amoxicillin, and pantoprazole. However, the results of these studies varied. Judaki, A. found that the addition of CCM increased the eradication rate of *H. pylori*. More RCTs are needed to confirm whether CCM can improve the eradication rate of *H. pylori* with triple therapy.

### 4.3. Colon Cancer

As previously stated, inflammatory bowel disease is a major contributor to colon cancer. CCM has been shown to alleviate the clinical symptoms of IBD by regulating inflammatory signals, thereby reducing the risk of IBD progressing to colon cancer. In addition, Mosieniak et al. [59] found that CCM induced cell cycle arrest and cell death in a concentration-dependent (0–40 μM) manner. CCM induces cell apoptosis by affecting the ratio of Bcl-2/Bax or Bcl-xL, upregulating DR5 protein, and activating caspase 8 to achieve its anticancer effect [60].

Shafei et al. [61] reviewed three RCTs and four quasi-experimental studies. The authors concluded that tumor size did not decrease when CCM was administered alone or in combination with other chemotherapy agents. Although CCM preparations result in significantly higher blood concentrations compared to traditional administration methods, the authors suggest that systemic blood levels are not a crucial factor in the treatment of colon cancer through oral administration. Instead, it is more reasonable to aim for appropriate local drug concentrations within the colon tissue. Two RCTs Table 1 [53,54] have shown that CCM supplementation improved biochemical markers in colon cancer patients compared with placebo. However, two RCTs did not report any changes in tumor size.

## 5. CCM Nanopreparation in Gastrointestinal Disease

Regulatory agencies, including the World Health Organization, allow a daily intake of up to 3 mg/kg of CCM [62]. However, even with high oral doses, plasma levels remain significantly lower due to its poor water solubility [63]. High doses of CCM can cause adverse reactions in the gastrointestinal tract when trying to achieve a certain blood concentration. Nanopreparation has become increasingly popular in recent years, and many drugs containing them are widely used in the pharmaceutical field. The size of Nanopreparation ranges from 1 to 1000 nm. Depending on the intended purpose, it can be designed in various ways, such as nanoparticles [64,65], liposomes [66], and nanoemulsions [67]. The emergence of nanopreparation offers promising prospects for CCM. Nanopreparation is expected to address the issues of CCM’s low bioavailability. The authors listed the CCM nanopreparations for gastrointestinal disease in Table 2.

### 5.1. Nanoparticle

Atoms or molecules gather under certain conditions to form stable small clusters (nucleation) and are continuously added to these clusters, which gradually grow into nanoparticles (growth) [79]. There are many types of nanoparticles, including polymer nanoparticles [13] and metal nanomaterials [80].

Han et al. [65] constructed a pH/ROS sensitive CCM nanoparticle in yeast particles. The author attached the dual-sensitive materials to β-cyclodextrin (β-CD), the D-mannose (Man) is modified to adamantane, and then loaded with CCM (Man-CUR NPs). The authors obtained nanoparticles with a particle size of 143.44 ± 2.05 nm and zeta potential was about +16.6 ± 0.21 mv. The in vitro pharmacologic research of Man-CUR NPs was conducted by using LPS-induced RAW264.7 macrophages. The concentration of pro-inflammatory cytokines (TNF-α, IL-1β) secreted by Man-CUR NPs-treated cells was significantly reduced compared to CUR NP-treated cells.

Sharma et al. [81] prepared CCM-loaded solid binary lipid nanoparticles (C-SBLN) with a high packing rate (83.12) and drug loading of 6.57% using stearic acid and tristearic acid as raw materials by continuously optimizing the formulation and process and adding surfactants such as Tweene-80. C-SBLN has good gastrointestinal stability and the drug release time is extended to 24 h. After oral administration of C-SBLNs to the DSS-induced colitis model, the biochemical indices of the model were significantly improved.

### 5.2. Liposome

Liposomes are formed by the spontaneous assembly of phospholipid molecules driven by hydrophobic and other intermolecular interactions [82]. The structure of the liposome is similar to the cell membrane, which makes the liposome highly biocompatible and biodegradable. In addition, the surface of the liposome can be modified to make it tissue-targeting [66].

Sesarman’s team has shown great interest in studying liposomes loaded with CCM and doxorubicin. The authors selected the anticancer drugs doxorubicin (DOX) and CCM encapsulated in liposomes, which greatly improved the anti-tumor efficacy. In addition, the authors found that pegylated long-circulating liposomes co-encapsulating CCM and DOX (LCL-CURC-DOX) had a strong anti-proliferation effect on mouse C26 colon cancer cells [83] and applied it to mouse colon cancer [66]. Sesarman et al. have provided sufficient evidence both in vivo and in vitro to prove that the therapeutic effect of LCL-CURC-DOX is much higher than that of free CCM, free DOX, CCM+DOX, and LCL-loaded CCM and DOX, respectively. In vivo, compared to free CCM (~190mm^3^) and DOX (~190mm^3^), treatment with LCL-CURC-DOX, significantly reduced tumor size (~50mm^3^).

### 5.3. Noisome

The structure of niosomes is similar to that of liposomes, both of which have a lipophilic bilayer structure, but the lipophilic property of niosomes comes from the non-ionic surfactant in the raw material.

Firouzi Amandi et al. [72] prepared CCM and artemisinin (Art) co-loaded niosome nanoparticles (Cur-Art NioNPs) and found that Cur-Art NioNPs could inhibit the proliferation of human colorectal adenocarcinoma cell SW480 by regulating apoptosis factors. Noisome can enhance the bioactivity of CCM and Art.

### 5.4. Micelle

In an aqueous solution, when the surfactant reaches the critical micelle concentration of the CMC, the surfactant spontaneously adsorbs together into a spheroid structure—micelle. At this point, the hydrophilic head end of the surfactant will extend outward and encapsulate the hydrophobic drug, greatly improving the solubility of the drug [82].

To improve the bioavailability of CCM, Gao et al. [73] obtained curcumin-supported polymer micelles (Cur/MPEG-PLA) with 8% drug loading using biodegradable mono-methoxy polyethylene glycol polylactide copolymer (MPEG-PLA). The authors demonstrated that the therapeutic effect of Cur/MPEG-PLA was much greater than that of free CCM in vitro and in mice with colon cancer. In addition, the effectiveness of Cur/MPEG-PLA therapy was evaluated on a mouse model of colon cancer. Cur/MPEG-PLA was found to have a greater inhibitory effect on colon tumor growth than free curcumin at the same dose (*p* < 0.05 or *p* < 0.05, respectively). This suggests that Cur/MPEG-PLA can enhance the antitumor effect of curcumin in vivo.

Furthermore, in another study in 2021, Hu et al. [74] took methoxy-poly(ethylene glycol)-poly(glycaprolactone) (MPEG-PCL) copolymer and added folic acid (FA) to the polymer structure to form FA/Nano-Cur. The therapeutic effect of CCM on colon cancer in mice was greatly enhanced by targeting folate receptors, which are highly expressed in malignant tumors. In vitro, FA/Nano-Cur and Nano-Cur induced more cell apoptosis than Free Cur at the same concentration. In vivo, the tumor-inhibition rate was 77.32% in the FA/Cur-Nano group and 16.25% in the Free CCM group on the 18th day. It was proved that FA/Nano-Cur has a stronger antitumor effect than free CCM in treating colon cancer.

### 5.5. Nanoemulsion

Nanoemulsion is a transparent/translucent and dynamically stable dispersion system formed from water, oil, and surfactants. Oil droplets can be dispersed in water to form an O/W (oil-in-water) emulsion or water droplets can be dispersed in oil to form a W/O (oil-in-water) emulsion. W/O emulsions increase drug solubility by coating hydrophobic drugs [84].

Lei et al. [75] prepared a Ph-responsive sodium alginate (SA) hydrogel-coated nanoemulsion that co-delivers CCM and emodin (EMO) CUR/EMO NE@SA, controls drug release in the colon, and specifically targets colonic macrophages. In preclinical studies, CUR/EMO NE@SA was shown to improve the inflammatory microenvironment of the colon by modulating inflammatory signaling and scavenging ROS from macrophages. The authors demonstrate that the nanoemulsion delivery system can significantly enhance the therapeutic effect of CCM and EMO. On activated macrophages, the levels of TNF-α and IL-6 were lower in CUR/EMO NE group than in free CCM group. In DSS-induced IBD, the DAI score of the CUR/EMO NE@SA group (~1) was lower than the free CCM group (~2). In addition, tissue inflammation levels were significantly reduced in the CUR/EMO NE@SA group. These results suggested that the CUR/EMO NE@SA could effectively ameliorate DSS-induced IBD.

Mosallam et al. [76] used a nanoemulsion system to combat *H. pylori* infection. The authors prepared a curcumin–clarithromycin nanoemulsion (Cur-CLR-NE) using an oil-in-water system. In vitro results showed that the MIC of Cur-CLR-NE was 6.26 μg·mL^−1^, and the MIC of free CCM was 50 μg·mL^−1^. The authors found that Cur-CLR-NE exhibited more potent antibacterial effects than free clarithromycin or CCM, including the clearance effect on *H. pylori* and the repair effect on damaged tissues. Both in vitro and preclinical studies have shown that Cur-CLR-NE has a stronger inhibitory effect on *H. pylori* than free CCM.

### 5.6. Organic and Inorganic Hybrid Nanopreparation

Organic polymers can improve the stability and biocompatibility of nano-delivery systems, and inorganic components help adsorb drugs and target diseases. This class of nanomaterials combines the advantages of organic polymers and inorganic materials to improve the functionality of nano-delivery systems and enhance drug efficacy.

Dhivya et al. [77] prepared CCM nanoparticles with polymethacrylate (PMMA) and polyethylene glycol (PEG) as the organic components, and ZnO as the inorganic component (PMMA-PEG/ZnO). Free CCM and CCM-supported PMMA-PEG nanoparticles were compared. The curcumin-loaded PMMA-PEG/ZnO nanocomposite has large observable effect on AGS cancer cell viability with an observed IC50 close to 0.01 μg·mL^−1^, while free CCM IC50 was approximately 0.05 μg·mL^−1^. PMMA-PEG/ZnO nanoparticles loaded with CCM showed better anti-gastric cancer cell activity.

Liu et al. [78] prepared aminated mesoporous silica support (MSN-NH2) and linked alginate oligosaccharides (AOS) on the surface of the support to obtain MSN-NH2-AOS nanoparticles. MSN-NH_2_-Cur-AOS nanoparticles were obtained by entrapping CCM in this carrier. The pH-sensitive AOS coating made the total release rate of Cur only 28.9 ± 1.6% under neutral conditions and 67.5 ± 1% under acidic conditions. In the same year, Jie Liu et al. [85] constructed a novel MnO_2_ shell nano platform, which used MnO_2_ to target CCM and DOX to tumor cell cancer molecules, achieving enhanced double chemotherapy of primary tumors and significant inhibition of distant colorectal tumors.

## 6. The Properties of Nanopreparations Affect the Release of Curcumin

It is expected to reduce the dose of oral CCM and alleviate its side effects by designing granules for lesions and controlling the drug release rate. Factors that influence the release rate of nanomaterials include particle size, zeta potential, and dispersity.

Lacob et al. [86] suggested that the drug release profile of the chitosan nanoparticle matrix is improved by reducing the size of the nanoparticles. Smaller nanoparticles have a higher specific surface area, and the dissolution rate of the drug will be higher [87].

In addition, Lombardo et al. [88] used the NanoDis system to measure the release profile of several nanoparticles. After comparing the results, we found that nanoparticle size and zeta potential can all affect drug release, but nanoparticle size appears to be the most important factor. Lombardo et al. showed that the release profile of PS80-C-high nanoparticles with the largest size (422.8 nm) was the worst and the release profile of PS80 nanoparticles with the smallest size (120.7 nm) was the best. However, the influence of the zeta potential is not insignificant. At similar size and PDI, P188 benchtop (251.7 nm, polydispersity index PDI = 0.10, zeta = −52.4 mV) had a better release profile than P188-C (254.4 nm, PDI = 0.09, zeta = 25.5 mV).

Stuart et al. [89] found that synthetic polydisperse population (where the variation in nanoparticle diameter is due to the synthesis process) and size-dependent protein corona polydisperse population (where nanoparticles are immersed in a protein-rich fluid and a protein corona forms on the surface of the nanoparticles) provided higher doses than monodisperse populations of the same average size.

## 7. Conclusions and Prospect

Gastrointestinal disease is a significant global health concern. Chronic inflammation of the gastrointestinal tract may lead to cancer. Traditional drugs have been the first choice for clinical treatment due to their proven therapeutic efficacy. However, the appearance of adverse reactions and drug-resistant bacteria lead clinical drugs to present challenges. CCM, a natural medicine, offers better safety than traditional medicine. Ample evidence supports the role of CCM in treating gastrointestinal disease, making it a promising supplement for prevention and treatment. However, further research is needed to fully understand its potential adverse reactions and mechanisms.

Additionally, the bioavailability of CCM requires continuous attention. The poor water solubility and stability of CCM in the intestinal tract pose challenges to its absorption and metabolism. However, researchers have discovered that the oxidative metabolism of CCM is necessary to inhibit the growth of *H. pylori*, as well as the translocation and phosphorylation of CagA and cSrc. Thus, the metabolism of CCM has both positive and negative effects. Its metabolites may target specific proteins and cytokines, while the biological activity of CCM may decrease after metabolism. Therefore, researchers should not solely concentrate on CCM, but also consider its metabolites as potential small molecule ligands for disease targets.

To enhance the bioavailability of CCM, researchers encapsulated it into nanoparticles, which can improve the solubility and therapeutic potential of CCM. Each property of the nanoparticles affects the release of CCM, and these properties (size, dispersion) need to be considered in the design of nanopreparations. However, questions remain regarding the absorption of the carrier and the potential side effects on the body. Additionally, some researchers propose that CCM’s drug concentration should focus on the gastrointestinal environment to target gastrointestinal cells, rather than its blood concentration. For the treatment of gastrointestinal disease, the author believes that future research on CCM nanopreparations should be more inclined to target the gastrointestinal tract.

It is worth noting that curcumin has been classified as both a pan-assay interference compound and an invalid metabolic panacea. CCM Keto-enol tautomer exhibits all known PAINS-type behaviors. Therefore, the biological activity of CCM really needs to be critically evaluated.

## Figures and Tables

**Table 1:** Summary of curcumin randomized controlled trials.

Disease	Study	Sample Size		Intervention		Age (years)		Duration
		Trial Group	Control Group	Trial Group	Control Group	Trial Group	Control Group	
UC	Hanai et al. [9,41,42] 2006	43	39	CCM 2000 mg/d + sulfasalazine 1000–3000 mg/d or mesalamine 1500 mg–3000 mg/d	Placebo + sulfasalazine 1000–3000 mg/d or mesalamine 1500 mg–3000 mg/d	45.2 ± 15.8	39.7 ± 14.2	24 weeks
	Singla et al. [9,41,43] 2014	14	16	CCM enema + 5-Aminosalicylic acid	Placebo enema + 5-Aminosalicylic acid	32.7 ± 8.9	35.5 ± 13.8	8 weeks
	Lang et al. [9,41,44] 2015	25	22	CCM 3000 mg/d + mesalamine	Placebo + mesalamine	40.4 ± 12.7	41.4 ± 13.9	4 weeks
	Kedia et al. [41,45] 2017	16	25	CCM 450 mg/d + mesalamine 2400 mg/d	Placebo + mesalamine 2400 mg/d	36 ± 12	34 ± 7	8 weeks
	Masoodi et al. [41,46] 2018	28	28	CCM 240 mg/d + mesalamine 3 g/d	Placebo + mesalamine 3 g/d	38.21 ± 16.37	36.04 ± 11.78	4 weeks
	Sadeghi et al. [9,41,47] 2020	31	32	CCM 1500 mg/d	Placebo	40.1 ± 13.2	40.6 ± 12.4	8 weeks
	Banerjee et al. [9,41,48] 2021	30	32	CCM 100 mg/d + mesalamine	Placebo + mesalamine	33.56 ± 10.1	34.66 ± 10.27	12 weeks
Crohn	Bommelaer et al. [9,41,49] 2020	31	31	CCM 3000 mg/d + 2–2.5 mg/kg/d azathioprine	Placebo + 2–2.5 mg/kg/d azathioprine	36.3 ± 8.9	32.9 ± 13.4	24 weeks
	Sugimoto et al. [9,41,50] 2020	17	9	CCM 360 mg/d	Placebo	36.3 ± 8.9	32.9 ± 13.4	12 weeks
*H. pylori* infection	Khonche et al. [51] 2016	30	30	CCM 500 mg/d + clarithromycin 500 mg/d + amoxicillin 1000 mg/d + pantoprazole 40 mg/d	Placebo + clarithromycin 500 mg/d + amoxicillin 1000 mg/d + pantoprazole 40 mg/d	35.03 ± 9.29	35.10 ± 8.96	4 weeks
	Judaki et al. [52] 2017	50	50	CCM 2100 mg/d + omeprazole 40 mg/d + amoxicillin 2000 mg/d + and metronidazole 1600 mg/d	Placebo + omeprazole 40 mg/d + amoxicillin 2000 mg/d + and metronidazole 1600 mg/d	53.65 ± 15.65	54.65 ± 16.54	4 weeks
Colon cancer	Panahi et al. [53] 2021	36	36	CCM (500 mg) + piperine (5 mg) capsule	Placebo capsule	58.68 ± 12.24	63.94 ±10.40	8 weeks
	Macis et al. [54] 2023	15	14	CCM (Meriva) 1000 mg/d + anthocyanin (Mirtoselect) 1000 mg/d	Placebo + anthocyanin (Mirtoselect) 1000 mg/d	70.8 ± 9.8	67.9 ± 10.8	4–6 weeks

**Table 2:** CCM nanopreparation for gastrointestinal disease.

Category	Study	Carriers	Size (nm)	Zeta-Potential (mV)	Polydispersity Index	Encapsulation Efficiency (%)	Drug Loading (%) (mg CCM/100 mg Polymer)	Disease Models	Drug Combination	Conclusion
Nanoparticle	Beloqui et al. [68] 2014	PLGA/Eudragit S100	116 ± 3	−40.4 ± 0.6	0.261 ± 0.03	67 ± 8	7.4 ± 0.9	Mouse colitis	NA	Compared with CCM, nano-CCM DSS-treated mice showed a significant reduction in both MPO activity and TNF-α secretion.
	Sufi et al. [69] 2020	PLGA/Tween-80	30–250	-	-	~74	7.4 ± 0.8	SW480 cell	NA	Drug loaded nanopreparation offers stability in different pH; Nano-CCM IC50 = 7–9 μM, CCM IC50 = 15–17 μΜ.
	Alam et al. [70] 2022	PLGA	175 ± 2.1	−16.4 ± 0.38	0.1 ± 0.004	80 ± 2.1	-	*H. pylori*	NA	CCM MIC = 16 μg·mL^−1^; nano-CCM MIC = 8 μg·mL^−1^.
	Chen et al. [71] 2022	P@HMPB ^1^	~173	~−14.7	-	~58.2	-	Mouse colorectal cancer	5-fluorouracil	5-Fu/Cur ^2^-P@HMPB group was found to have the highest tumor inhibition efficiency.
	Han et al. [65] 2023	β-CD/Man/YPs	143.44 ± 2.05	+16.6 ± 0.21	-	90.24 ± 1.49	-	Mouse colitis	NA	After the treatment with Man-CUR ^2^ NPs, the level of TNF-α and IL-1β is significantly lower compared to the free CCM.
Liposome	Sesarman et al. [66] 2019	PEG-LEL	170	−50	<0.1	>90	-	Mouse colon cancer	Doxorubicin	Compared to free CCM (~190 mm^3^) and DOX (~190 mm^3^), treatment with LCL-CURC-DOX significantly reduced tumor size (~50 mm^3^).
Noisome	Firouzi et al. [72] 2023	Span 80/Tween 80/Cholesterol	210.10 ± 13.04	−50.47 ± 0.47	0.47 ± 0.08	93.36 ± 0.10	-	SW480	Artemisinin	Compared with free CCM and Art, treatment with Cur ^2^-Art NioNPs significantly inhibit the growth of SW480 cells.
Micelle	Gao et al. [73]. 2013	MPEG-PLA	~30	-	-	-	8.0	Mouse colon cancer	NA	The therapeutic effect of Cur ^2^/MPEG-PLA was much greater than that of free CCM in vitro and in mice with colon cancer.
	Hu et al. [74] 2020	MPEG-PCL	30.47 ± 0.65	−3.55	0.17	98	-	CT26 cells; Mouse colorectal cancer	FA	FA/Nano-Cur ^2^ and Nano-Cur ^2^ induced more cell apoptosis than Free CCM at the same concentration. In vivo, tumor size grew slowly in the treatment with FA/Nano-Cur ^2^.
Nanoemulsion	Lei et al. [75] 2023	hydrogel/SA ^3^	130.4 ± 2.4	−21.6 ± 1.9	<0.3	88.75 ± 1.82	2.36 ± 0.04	Mouse IBD	Emodin	the levels of TNF-α and IL-6 were lower in CUR ^2^/EMO NE group than in CCM group.
	Mosallam et al. [76] 2023	coconut oil/Tween 80/Propylene glycol	61.2 ± 2.15	+0.57 ± 4.05	0.245 ± 0.01	98 ± 2	-	Mouse infected with *H. pylori*	Clarithromycin (CLR)	Cur ^2^-CLR-NE MIC = 6.25–12.5 μg·mL^−1^; Free CCM MIC = 50 μg·mL^−1^.
Organic and inorganic hybrid nanopreparation	Dhivya et al. [77] 2017	PMMA-PEG/ZnO	40–90	-	-	-	~92	AGS cell	NA	PMMA-PEG/ZnO nanoparticles loaded with CCM showed better anti-gastric cancer cell activity.
	Liu et al. [78] 2022	MSN-NH2-AOS ^4^	150.8 ± 4.6	−32.2 ± 0.6	0.190 ± 0.039	91.24 ± 1.23	-	HCT-116 cell	NA	The pH-sensitive AOS ^4^ coating made the total release rate of Cur only 28.9 ± 1.6% under neutral conditions and 67.5 ± 1% under acidic conditions. the MSN-NH2-Cur ^2^-AOS ^4^ nanoparticles were more easily absorbed by colon cancer cells than free CCM, achieving a high tumor cell targeting efficiency.

^1^ Prussian blue; ^2^ CCM; ^3^ Sodium alginate; ^4^ Alginate oligosaccharides.
